# Leucoma of the Vulva

**Published:** 1901-07-20

**Authors:** 


### LEUCOMA OF THE VULVA.

MR. BUTLIN[1] points out that the areas of more or less horny epithelium which are sometimes met with on the vulva, just as they are on the buccal mucous membrane, are apt to be associated with cancer. These conditions of leucoma or leucoplakia of the vulva have hitherto received but little attention in this country, but they are probably of some importance as a predisposing cause of cancer of the vulva. He describes several cases. A maiden lady 44 years of age was seen in April. She was then troubled with a peculiar condition of the vulva. There were numerous white areas covering a large part of the surface of the greater and lesser labia. Some were thin and bluish-white, others were thick and opaque. They strongly resembled the plaques which are so frequent on the surface of the tongue and which are now classed together as the result of chronic superficial inflammation. The patient had only noticed this condition recently, but it might have been there for months, or years, for all she could tell to the contrary. In July of the same year she had an indurated ulcer at the upper part of the right labium majus, in the midst of the white plaques. The ulcer had all the appearance of epithelioma in an early stage, and arrangements were made for its removal. The operation however did not take place, and Mr. Butlin heard later that six months after he had seen the patient she died of some kind of debility and anæmia. Another case was that of a widow lady, aged 73, who was suffering from a circular ulcerated epithelioma of the right labium majus. The ulcer was situated in a plaque of very thin white leucoma, and there were similar areas which covered all the upper part of both labia on both sides, and the central portion of the vulva about the orifice of the urethra and the clitoris. She did not know of their existence and had apparently suffered no inconvenience from them until the cancer had commenced to form. The cancer was removed freely and the plaques were dissected away. Another case is given in which again this condition of leucoma of the vulva was associated with the occurrence of cancer in the same part and secondarily in the glands of the groin. These leucomatous patches are on the mucous surfaces only and not on the skin; they are precisely similar in appearance, feel, and variety of form to the white plaques which form upon the mucous surface of the mouth, and in more than one of the recorded cases the vulva and the mouth have been attacked in the same patient. There would seem, therefore, to be much reason to believe that it is the same disease in both situations; and if so one must evidently reconsider much that has been laid down as to the influence of tobacco and of the direct contact of alcohol in the production of these white patches in the mouth.

[1] Brit. Med. Journ., July 13.

